# Public's perceptions and attitudes toward HIV and people living with HIV in GCC countries: a qualitative study

**DOI:** 10.3389/fpubh.2025.1718112

**Published:** 2026-01-09

**Authors:** Noura Alomair, Samah Alageel

**Affiliations:** Department of Community Health Sciences, College of Applied Medical Sciences, King Saud University, Riyadh, Saudi Arabia

**Keywords:** GCC, HIV/AIDS, people living with HIV, PLHIV, qualitative research, stigma

## Abstract

**Background:**

Despite advances in HIV treatment, stigma and discrimination toward people living with HIV (PLHIV) remain widespread across the Gulf Cooperation Council (GCC) countries. Limited qualitative research has explored both public attitudes and the lived experiences of PLHIV in the region.

**Methods:**

This qualitative study used semi-structured interviews with twenty-seven participants from five GCC countries, including PLHIV and members of the public. Data were analyzed using reflexive thematic analysis.

**Results:**

Findings showed that HIV stigma is driven by moral judgments, misinformation, and negative media representation. PLHIV reported experiences of social exclusion, fear of disclosure, and barriers to healthcare, education, and employment. Stigma was gendered, with women facing more severe social consequences. Participants highlighted the need for increased public awareness, improved media narratives, and specific policy reforms, including stronger confidentiality protections, non-discriminatory access to healthcare, and workplace safeguards for PLHIV.

**Conclusion:**

Reducing HIV stigma in the GCC requires coordinated, multi-level strategies that directly address the structural and social factors identified in this study. These include the development of stigma-free and integrated healthcare pathways, strengthened legal protections, and sustained public awareness efforts. The findings emphasize the importance of grounding policy and educational interventions in the lived realities of PLHIV and the broader public.

## Introduction

Human immunodeficiency virus (HIV) infection remains a significant global public health issue. According to the World Health Organization (WHO), over 39 million people globally were living with HIV in 2023 (1). While the overall incidence and mortality rates of HIV have demonstrated a downward trend since 2010, primarily due to enhanced access to testing and treatment interventions, this decline has not been geographically uniform (1, 2). Notably, the Middle East and North Africa (MENA) region experienced a 116% increase in new HIV infections from 2010 to 2023 (3). This rise represents the largest proportional increase among regions globally and signals a growing public health challenge. The sharp upward trend reflects persistent gaps in early testing, limited access to prevention services, and the ongoing impact of stigma on timely care seeking (4, 5).

Despite significant progress in the global HIV response, such as expanded access to antiretroviral therapy (ART) and a decline in new infections worldwide, stigma and discrimination related to HIV remain a significant issue worldwide (4, 5). In the MENA region, as with many parts of the world, criminalization and societal stigma continue to undermine effective responses to the HIV epidemic (6). Stigma and discrimination have a profound negative impact on the physical and mental health of people living with HIV (PLHIV) (7, 8).

Research has shown that stigma and discrimination occur at multiple levels, including internalized (self-stigma), interpersonal (social stigma), and institutional stigma, particularly within healthcare settings (5). This multilayered stigma acts as a barrier to prevention, diagnosis, and treatment services, ultimately impeding efforts to control the epidemic (6). These levels of stigma do not operate in isolation; instead, they reinforce one another in ways that shape broader community perceptions of HIV (9). Internalized fears and shame are often influenced by interpersonal reactions such as family rejection or discriminatory behavior, which themselves are shaped by structural factors including health system practices, media portrayals, and policy environments (9, 10). This interconnected stigma creates a cycle that normalizes negative attitudes toward HIV and solidifies stigma across social and institutional settings.

The Gulf Cooperation Council (GCC) countries, which include Bahrain, Kuwait, Oman, Qatar, Saudi Arabia, and the United Arab Emirates, are primarily Muslim and high-income nations located in the MENA region. While HIV prevalence in the GCC remains low compared to global figures, incidence rates have been steadily rising (11). Despite the availability of free, high-quality HIV services, the rates of early diagnoses and viral suppression remain suboptimal (11). This gap may be attributed to persistent HIV-related stigma and challenges in treatment adherence (4, 11). In Saudi Arabia, key barriers to testing and diagnosis include limited public knowledge, fear of stigma and judgment, concerns about confidentiality, and mistrust in the doctor–patient relationship (12). In the GCC countries, negative attitudes toward HIV are widespread, not only affecting the general public but also alarmingly present among healthcare professionals, who play a crucial role in addressing the issue (13). Such biases can hinder effective communication, treatment, and support for individuals living with HIV, further exacerbating the challenges faced by affected individuals. These individual and societal barriers continue to impede efforts to curb the spread of HIV, limiting the impact of governmental initiatives aimed at improving prevention, diagnosis, and treatment in the region.

Understanding the individual, societal, and institutional factors that drive stigma and discrimination against PLHIV is critical. These perceptions shape how communities interpret HIV risk, influence willingness to seek testing, and determine whether individuals access care in a timely manner. Evidence from regions such as South Asia and sub-Saharan Africa shows that stigma-reduction campaigns, community-led education and normalization of HIV testing have directly contributed to earlier diagnosis, increased antiretroviral therapy uptake and measurable declines in new infections, highlighting how shifts in community perception can transform HIV outcomes (14–16). To capture both societal attitudes and how these attitudes are experienced by those directly affected, the study included members of the public as well as individuals living with HIV. Exploring these complex dynamics can deepen existing knowledge and inform the development of more effective policies and practices within the GCC region and other societies with comparable cultural and structural contexts. Accordingly, this study aimed to explore public perceptions and attitudes toward HIV and individuals living with HIV in the GCC countries.

## Methods

This study employed a qualitative research design to explore public perceptions and attitudes toward HIV and individuals living with HIV in the GCC countries. It was grounded in an interpretivist qualitative paradigm, which recognizes that meanings, attitudes, and social perceptions are constructed through cultural, religious, and interpersonal contexts and are therefore best understood through in-depth engagement with participants' lived experiences (17).

A semi-structured interview format facilitated open-ended discussions and elicited in-depth insights into participants' views and experiences. This approach was chosen for its capacity to capture the complexity of social attitudes and to provide an interpreted understanding of participants' beliefs within their cultural context (18). Understanding the attitudes and beliefs held by the public regarding HIV and PLHIV is essential in tackling the various challenges surrounding HIV prevention, both in the region and worldwide. Qualitative methods are particularly well-suited for examining sensitive topics such as HIV, as they facilitate rich, contextualized data collection that can reveal underlying social and structural barriers to effective prevention and care (19).

This study was reviewed and approved by the King Saud University Human Research Ethics Committee (approval number: KSU-HE-21-151). It is part of a larger project exploring multiple aspects of sexual health in the GCC (20, 21).

## Sampling and recruitment

A purposive sampling strategy was used to recruit individuals from diverse geographical areas across the GCC, representing various age groups and including both male and female participants. The study targeted adults aged 18 years and above residing in the GCC, as well as individuals living with HIV. Despite the sensitivity of the topic, PLHIV expressed strong interest in participating, explaining that they wanted their voices to be heard and their experiences shared, and viewed anonymous participation as an opportunity to contribute to a greater understanding of their lived experiences with HIV stigma.

Recruitment was conducted through social media platforms, primarily via X (formerly Twitter), where an invitation post outlined the study's objectives and included a link to the participant information sheet and electronic consent form. Individuals who expressed interest completed the consent form and indicated their preferred method of contact. SA followed up using the selected communication method (email or phone) to schedule the interview.

## Data collection

Before each interview, participants completed a brief demographic survey to collect descriptive data, including gender, age, education level, employment status, and country of residence. Interviews were conducted online via Zoom, enabling participation from individuals across the GCC region and providing a private and comfortable environment for discussing a highly sensitive and stigmatized health condition such as HIV. Conducting the interviews virtually also reduced potential embarrassment, fear of recognition, or social pressure that can arise in face-to-face settings when discussing taboo topics.

All interviews were conducted by SA, a Saudi female public health researcher with training and experience in qualitative research methods. With participants' informed consent, interviews were audio-recorded for transcription and analysis. Data collection occurred between March and April 2021, with interviews lasting 20–60 min.

The HIV Stigma Framework, developed by Earnshaw and Chaudoir, was used to guide this study, offering a comprehensive model for understanding stigma from the perspectives of both PLHIV and the general public, aligning well with the composition of our sample (9). The framework distinguishes three mechanisms of stigma experienced by PLHIV: internalized stigma (negative beliefs about oneself), anticipated stigma (expectation of future discrimination), and enacted stigma (experiences of actual discrimination) (9). Differentiating between these mechanisms is crucial for understanding how stigma affects psychological wellbeing, social relationships, and access to healthcare and other essential services. The framework also outlines how stigma is manifested among the general public through prejudice, stereotyping, and discrimination toward people living with HIV (9). It emphasizes the influence of broader social norms, cultural values, and institutional structures in shaping these attitudes and behaviors, which can contribute to the marginalization and reduced quality of life for individuals affected by HIV. Using this model enabled the study to sensitively explore how social norms, cultural values, and institutional structures shape HIV-related attitudes in the GCC.

A semi-structured topic guide was developed based on this framework and a review of literature examining perceptions of HIV and sexually transmitted infections in Muslim-majority contexts (4, 12). Given the stigmatized nature of HIV, the semi-structured interviewing approach provided participants with the flexibility to explore sensitive topics at their own pace. This provided them with space for reflection and emotional expression, while ensuring that core issues were consistently addressed.

The guide included questions on participants' understanding of HIV transmission, perceptions of people living with HIV, and views on appropriate interventions for the GCC context. It also explored factors participants believed contributed to HIV infection and strategies for improving the lived experience of people diagnosed with HIV. The topic guide was piloted with one participant and refined based on feedback before formal data collection began. Participants were encouraged to raise additional topics not explicitly covered by the guide.

## Data analysis

All interviews were transcribed verbatim, and a reflexive thematic analysis approach was employed (22). Data were managed and coded using ATLAS.ti software. An inductive coding strategy was adopted, with SA conducting the initial coding. The first author, NA, a Saudi female public health researcher with expertise in sexual and reproductive health and qualitative research, independently double-coded a subset of interviews.

The research team held regular meetings throughout the analysis to review and refine the coding process. Initial codes were organized into a preliminary thematic framework, which the authors iteratively reviewed and adjusted to ensure it accurately reflected the data. Themes and sub-themes were developed collaboratively through discussions between authors to enhance the rigor and credibility of the findings (19).

The interviews were conducted in Arabic, and the transcripts were analyzed in Arabic through a systematic coding process to preserve the original meaning and cultural context of participants' narratives. The illustrative quotes included in the findings were translated into English by NA and SA, after which both authors reviewed and agreed on the final translations to ensure semantic accuracy and contextual integrity.

## Results

Twenty-seven participants took part in this study. We had a diverse sample of male and female participants aged from 18 to 50 years old. Two of the study participants are living with HIV. Four themes were identified from the data, including *Perceived Drivers and Patterns of HIV Transmission in GCC Contexts, Stigma toward HIV/AIDS, The lived experiences of people living with HIV and The personal and professional implications of living with HIV*.

## Theme 1: perceived drivers and patterns of HIV transmission in GCC contexts

### “Not our disease”: perceived immunity and denial

Many participants commented on the prevalence of HIV/AIDS in the GCC, often attributing its presence, or the behaviors believed to cause it, to influences from Western societies. There was a widespread view that HIV/AIDS was uncommon, with participants highlighting religion and conservative norms in protecting locals. Some saw the GCC as largely immune due to its Islamic values and social norms restrictions.

“*These things don't exist here because we are Muslims and live in a conservative society. So this doesn't happen here often, unlike in the West.”* **(P26, Female, 34-40 years old, Kuwait)**

The perception that HIV/AIDS is not “our” disease was shaped by the belief that behaviors commonly associated with its transmission, such as extramarital sex, are more prevalent among non-Muslims. Many participants perceived GCC societies as inherently safeguarded by religious and social norms, which were viewed as barriers to the spread of HIV. This sense of cultural and moral immunity reinforced the idea that HIV is fundamentally a foreign issue.

“*I think GCC countries have it [HIV cases] way less than other countries. Given our religion and societal circumstances.”* **(P27, Male, 41-50 years old, Bahrain)**

“*It's mainly prevalent among non-Saudis and individuals who are unable to afford marriage and lack morals*.” **(P3, Male, 26-33 years old, Saudi Arabia)**

Although many participants expressed a sense of cultural immunity, others simultaneously questioned the accuracy of reported HIV prevalence, reflecting different but coexisting ways of understanding HIV in the region. They doubted official figures, suggesting the accurate scale might be underreported due to cultural sensitivities and stigma.

“*It might be prevalent, but I don't really know. We don't have any statistics, so I cannot give you an informed answer. These numbers are hidden from us.”* **(P14, Male, 41-50 years old, Kuwait)**

One participant explained that HIV/AIDS is under-recorded in GCC countries due to a lack of diagnosis.

“*Sadly, I believe the actual numbers are much higher due to underreporting from lack of testing.”* **(P20, Male, 18-25 years old, Kuwait)**

### Linking HIV to moral decline

Participants identified the increasing consumption of pornography and engagement in prostitution as key contributors to the rise in premarital sex, which they viewed as a major driver of HIV/AIDS transmission. Easy access to the internet was believed to facilitate exposure to pornography across all age groups, contributing to what some described as a moral decline in society. These concerns were often linked to delays in the age of marriage and a perceived lack of effective social or governmental interventions.

“*We need to find a solution for the delay in the age of marriage. The availability of pornography is a very serious, dangerous, and damaging issue in our community. It's widespread and may God protect us from these things, but how can we control it?”* **(P16, Male, 41- 50 years old, Kuwait)**

Participants frequently cited the absence of family support and weakening of religious values as contributing factors to the rise in extramarital relationships, which were viewed as key pathways for HIV/AIDS transmission. Participants believed that individuals with strong familial ties and a foundation in religious and moral teachings were less likely to engage in behaviors that increase the risk of infection. Additional influences mentioned included peer pressure, lack of awareness, and the growing impact of the internet and social media on youth behavior.

“*First of all, lack of family support, because as you know, if the family is not looking after their children, they will stray. Second, peer pressure plays a role. Third, lack of awareness or truancy. Lastly, the internet and social media have become recent factors.”* **(P14, Male, 41-50 years old, Kuwait)**

### Broader perceptions of HIV transmission

In addition to extramarital sex, participants identified several other modes of HIV transmission. These included intravenous drug use, unlicensed medical procedures, and steroid or cosmetic injections. Some also expressed concerns about unsafe practices in non-regulated clinics locally and abroad. Some participants associated HIV/AIDS with homosexuality, believing men who have sex with men were especially at risk.

“*It could be caused by drug use, using a used needle. However, it's important to note that the needle must have been used within the last 10 minutes. Other causes could include unlicensed cupping, unlicensed clinics, or cosmetic injections.”* **(P20, Male, 18-25 years old, Kuwait)**

“*It comes from men who have sex with men. From practices that are between men, which makes it more likely for this to be prevalent among men.”* **(P9, Female, 34-40 years old, Kuwait)**

### Gendered risk and societal double standards

Most participants perceived males in GCC countries as more susceptible to HIV/AIDS, primarily due to their greater social freedom and increased likelihood of engaging in high-risk behaviors, such as extramarital sex and drug use. Within the conservative context of the GCC, men are generally afforded more autonomy and face fewer social consequences for behavior that contradicts cultural and religious norms.

“*I must admit, being male here is synonymous with more freedom. They have much more freedom than females, allowing them to travel and visit places with a higher risk of engaging in risky behaviours. On the other hand, females in the GCC have less freedom due to cultural values and beliefs.”* **(P24, Male, 18-25 years old, Oman)**

Assumptions about the source of infection often shaped perceptions of HIV/AIDS. In the GCC context, participants identified several behaviors believed to contribute to the spread of the virus, with a particular emphasis on men's mobility. The term “traveling” was frequently used as a euphemism to imply male involvement in extramarital sex while abroad.

“*I think extramarital relationships are common among men, especially when they travel. We don't have these practices here in Saudi Arabia, so men usually engage in them when they travel.”* **(P11, Male, 26-33 years old, Saudi Arabia)**

Participants highlighted a clear gendered double standard regarding extramarital sex. Women are expected to abstain from sexual activity before marriage, and those who deviate from this norm are often judged more harshly than men who engage in the same behavior. This disparity was seen as rooted in deeply ingrained cultural and societal norms.

“*In our society, men can do no wrong. If it were a girl, she's a degenerate, and she caused it to herself. They would attribute it to a lack of awareness if it were a man. Whereas if it were a woman, she didn't just make a mistake, she committed a shameful sin.”* **(P22, Female, 18-25 years old, Oman)**

Despite the perception that men are more likely to engage in extramarital affairs, women were often held responsible for such behavior. Some participants suggested that women's attire contributed to men's infidelity, based on the belief that revealing clothing provokes men's instincts. It was also noted that men were perceived to have limited or no control over their sexual desires.

“*This Westernised view of feminism and women's equality is incorrect. Women have their role in society, but that doesn't mean they can befriend men and socialise with them. If something were to happen, it should be within the bounds of a marital relationship and nothing else. This, I feel, is one of the reasons why it's more prevalent now.”* **(P16, Male, 41-50 years old, Kuwait)**

Some participants believed that the stricter social expectations placed on women in the GCC were beneficial, as they help protect women from acquiring HIV. These restrictions, which limit women's mobility and social interactions, were seen as reducing exposure to high-risk situations.

“*Our society imposes more restrictions on women than on men... While these restrictions are negative, they also reduce the likelihood of women contracting certain infections.”* **(P17, Male, 18-25 years old, Oman)**

Many participants stressed the importance of acknowledging that anyone is susceptible to HIV/AIDS, rather than attributing susceptibility to specific populations and outliers. However, when reflecting on their own lives, most did not perceive themselves or their families as being at risk of contracting HIV/AIDS.

“*I don't feel like people around me, including young men, are engaging in risky behaviours, so I feel they don't need to be educated about this.”* **(P6, Female, 26-33 years old, Kuwait)**

## Theme 2: stigma toward HIV/AIDS

Stigma surrounding HIV/AIDS and PLHIV was evident throughout participants' accounts. In some cases, participants directly expressed stigma; in others, it was described as a widespread attitude within the broader GCC community. Expressions such as “God protect us” or “God forbid” were commonly used when referring to HIV/AIDS, indicating fear and moral judgment.

### Factors contributing to HIV/AIDS stigma

Stigmas were often associated with the mode of transmission. Participants explained that because HIV/AIDS is frequently linked to “forbidden relationships,” it is viewed negatively. PLHIV were shamed for their diagnosis based on the assumption that they got it through extramarital sex. Participants explained feeling disgusted by PLHIV. Linking HIV/AIDS to immorality was seen to cause social ostracism for PLHIV.

“*We are very ignorant when it comes to this disease [HIV]. We believe that forbidden relations transmit it. If a respectable person ever had it, would society look at him the same? No, he will be destroyed. You know why? Because society will judge him and say, ‘How did you get this?' And then this will cause what you call social execution*.” **(P16, Male, 41-50 years old, Kuwait)**

HIV stigma was closely tied to religious and moral interpretations of the disease. Some participants viewed the condition as a form of divine punishment. This belief contributed to a lack of sympathy toward PLHIV, reinforcing the idea that they are responsible for their diagnosis and should suffer the consequences.

“*It is [HIV] directly connected to sex. It's a shameful disease, and the person who has it commits heinous acts, and God punished him and his family for it.”* **(P18, Female, 41-50 years old, United Arab Emirates)**

Stigma surrounding HIV/AIDS appeared to be deeply embedded in cultural perceptions, affecting PLHIV. The stigma led PLHIV to feel the need to distance themselves from those behaviors to avoid judgment. For example, one participant living with HIV emphasized that his infection was not the result of drug use or extramarital sex, but rather from unlicensed cupping.

“*I contracted it from unlicensed cupping. It doesn't have to be drugs or forbidden relations. There are many causes…”* **(P20, Male, 18-25 years old, Kuwait)**

Misconceptions and a lack of accurate knowledge were also identified as key contributors to the stigma surrounding HIV/AIDS. Some participants expressed fear of contracting the virus through casual contact, such as touching or being near someone living with HIV.

“*I feel like just touching or being close to someone with HIV can transmit the disease. I wouldn't dare talk to someone with HIV because I'm scared for myself; I'm scared I would get it.”* **(P4, Female, 26-33 years old, Saudi Arabia)**

Several participants identified media depiction of HIV/AIDS as a key source of stigma surrounding HIV/AIDS. Participants noted that HIV/AIDS is often portrayed in the media as a frightening and fatal disease, reinforcing fear and negative perceptions. Furthermore, PLHIV are frequently depicted as immoral or socially isolated, rather than as ordinary people living everyday lives.

“*Movies often depict people with HIV in a very negative light, which has deeply influenced the community's perception. This portrayal leads people to view individuals with HIV as bad, sinful, and deserving of their fate.”* **(P19, Female, 34-40 years old, Saudi Arabia)**

### Challenging stigma through awareness

Participants agreed that raising awareness of HIV/AIDS is essential to reducing stigma. They emphasized the need for awareness efforts that reach both the general public and healthcare professionals, noting that misconceptions and fear exist even among nurses and doctors. For participants, “inclusive” awareness meant campaigns that normalize HIV, highlight the rights and wellbeing of PLHIV, and involve everyone rather than focusing only on affected individuals or high-risk groups. Normalizing the disease and recognizing that PLHIV can live fulfilling lives were seen as key steps toward shifting negative perceptions.

“*We need to recognise the rights and wellbeing of PLHIV. The prevailing perception is that if you have HIV, your life is over, you will never have a fulfilling life, and you will never be able to get married. Even nurses are afraid to interact with them. There needs to be widespread awareness that involves healthcare professionals such as doctors and nurses, and everyone.”* **(P3, Male, 26-33 years old, Saudi Arabia)**

Participants suggested that individuals living with HIV/AIDS should take an active role in raising awareness and reducing the stigma associated with HIV/AIDS. They were viewed as the most effective advocates for presenting information and educating others about HIV/AIDS.

“*You should consider involving PLHIV to be a part of raising awareness and changing the community's perceptions toward the disease. It will capture their attention more than if the information came from a medical doctor*.” **(P10, Female, 26-33 years old, Saudi Arabia)**

Some participants expressed doubt about the effectiveness of awareness campaigns in reducing stigma. They believed that stigma runs deeper than misinformation and is rooted in broader societal attitudes.

“*To me, PLHIV are not a concern. If they take their medications and the viral load is low, they should live normally. But that's not the reason society rejects them; it's much deeper than that.”* **(P25, Male, 26-33 years old, United Arab Emirates)**

## Theme 3: the lived experiences of people living with HIV

### Initial reactions to HIV diagnosis

When PLHIV reflected on their experiences upon learning about their diagnosis, they initially reacted with fear and worry that their lives would be cut short. HIV/AIDS is commonly viewed as fatal, so people's initial response to the disease is the belief that it will end their lives.

“*Before I was diagnosed, I truly believed that it was a deadly disease. I also thought it could be transmitted through touch. When I first heard I had it, I thought I only had two weeks left before I died.”* **(P20, Male, 18-25 years old, Kuwait)**

PLHIV described gradually coming to terms with their diagnosis after learning more about the prognosis and available treatment. While they have adapted to living with the condition, participants expressed that society has not adapted in return. Participants, particularly PLHIV, called for the humanization of PLHIV and for representing them as ordinary individuals capable of leading healthy, productive lives.

“*I really wish that people would focus on the fact that individuals living with HIV look like everyone else and can lead normal, productive lives. They do not threaten the community by existing, especially if they adhere to their treatment. Unfortunately, the community refuses to accept this.”* **(P25, Male, 26-33 years old, United Arab Emirates)**

### Desire for normalcy and acceptance

Participants expressed mixed views on recent media campaigns that aimed to present HIV/AIDS as a manageable chronic condition, similar to diabetes or hypertension. While some saw these efforts as a way to reduce stigma and promote acceptance, others viewed them critically. They believed that portraying HIV/AIDS as a normal illness might encourage behaviors they considered socially unacceptable, such as extramarital sex, by downplaying the seriousness of the condition.

“*Why are they trying to make this look like diabetes or hypertension? Just take medication and you'll be fine! This has to stop*!” **(P18, Female, 41-50 years old, United Arab Emirates)**

### Barriers to disclosing HIV status

The social exclusion of people living with HIV/AIDS affected their willingness to disclose their diagnosis. Participants living with HIV expressed fear and hesitation about sharing their status, even with close family members. Some participants who are not living with HIV voiced disapproval of efforts to normalize open discussions about HIV/AIDS, viewing such openness as inappropriate. These attitudes contribute to ongoing fears of exposure and social judgment among PLHIV.

“*Many PLHIV choose not to disclose their diagnosis to their families. This decision is not due to shame. As Muslims, we believe in fate and destiny, so most individuals have come to terms with their situation. The primary concern is societal stigma and the fear of non-acceptance. Even those closest to them, including their own families, struggle to come to terms with the diagnosis*.” **(P25, Male, 26-33 years old, United Arab Emirates)**

Participants with HIV compared the stigma in the GCC to Western countries. Western societies were seen as more tolerant and less judgmental of behaviors like extramarital sex, viewing them as personal choices. In contrast, the GCC was described as oppressive for PLHIV. Consequently, PLHIV considered migration as a way to escape stigma and find a more supportive environment.

“*How can I be expected to live in a community that sees me this way? I feel very oppressed. Migration seems like the best option to escape from this brutal world... I used to never think about leaving Gulf society, but now I am focused on building a life abroad. When you ask me what our society is missing, I ask you: what aren't we missing? The community does not allow us to live and coexist peacefully.”* **(P20, Male, 18-25 years old, Kuwait)**

### The social and psychological impact of HIV diagnosis

The stigma around HIV/AIDS severely affects those with the condition. They are often marginalized, mistreated, or even isolated by family. This exclusion hampers their ability to form or sustain relationships, including friendships and marriage.

“*I can't get married, I can't tell a girl I want to get married, and I have this [HIV]. Impossible! She will definitely say no; she might tell her family why she said no, and her family might talk about me. If people find out about me, they avoid me, and I might commit suicide.”* **(P20, Male, 18-25 years old, Kuwait)**

The social exclusion and stigma experienced by people living with HIV/AIDS have a profound negative impact on their mental health. Participants noted that the psychological burden is often linked more to societal attitudes and stigma than to the medical condition itself. In some cases, the emotional toll of being ostracized led to feelings of hopelessness and thoughts of suicide.

“*People reach the point of wanting to commit suicide because of social exclusion and ostracism. As a person living with the disease, you will eventually accept it, but you can't control the views of people around you, and that makes the person feel like an outcast.”* **(P25, Male, 26-33 years old, United Arab Emirates)**

### Mental health and wellbeing of individuals living with HIV

People living with HIV/AIDS require psychological support to cope with the challenges associated with stigma in their communities. Participants living with HIV expressed the need for mental health care to be integrated into the medical services provided to them.

“*The government should require the Ministry of Health to provide PLHIV with mental health support. I'm grateful I had the resources to compose myself and heal mentally, but not everyone does. Many people have been living with depression and desperation because of their diagnosis for so many years.”* **(P20, Male, 18-25 years old, Kuwait)**

Stigma and fear surrounding HIV/AIDS have also created barriers to accessing testing and treatment. Participants highlighted that fear of judgment and the negative image of the disease contribute to delays in seeking testing. Providing anonymous and confidential testing services was viewed as essential to improving early diagnosis and access to care in GCC countries. When asked about reasons for delayed testing, one participant explained:

“*It's the fear, the fear, the fear, and the negative image of the disease. Other reasons include a lack of awareness. Many people have it and are not aware of it.”* **(P20, Male, 18-25 years old, Kuwait)**

### Coping strategies and resilience among individuals living with HIV

Due to the exclusion faced by people living with HIV/AIDS, social media platforms have become the only “safe space” where they can openly discuss their condition and seek support. In the GCC, the primary source of emotional and informational support comes from others who are also living with HIV/AIDS. Using pseudonyms, individuals connect through online platforms to form support networks with others across Arab countries. Although participants living with HIV preferred real-world relationships, concerns about confidentiality remain a significant barrier to in-person interaction.

“*We are unable to talk to anyone in person. The only way for us to express ourselves and seek support as PLHIV is through Twitter. Some social media accounts exist to support those living with HIV through shared experiences. I was recently diagnosed, and I desperately needed someone to talk to. A person contacted me privately on Twitter and explained the medical aspects of the disease and the importance of medication adherence. He gave me advice, including not telling anyone about my diagnosis. He truly helped me so much, and if Twitter was not there, I don't know if I would be here today.”* **(P20, Male, 18-25 years old, Kuwait)**

## Theme 4: the personal and professional implications of living with HIV

### Rules and regulations

The pervasive stigma surrounding people living with HIV/AIDS has had a profound impact on their ability to assert their rights. PLHIV expressed a deep sense of powerlessness when it comes to filing complaints or advocating for themselves, particularly in interactions with government institutions, due to the fear of being exposed and shamed for their diagnosis. They voiced concerns about the potential repercussions of disclosing their HIV status, including being denied access to healthcare and facing social ostracism. This fear has created a barrier for individuals living with HIV, impeding their ability to access the support and care they need.

“*The issue we face as PLHIV is that when we experience neglect or encounter any problems, we can't speak up. If a doctor mistreats you, raising concerns is very difficult because doing so would expose us. Sadly, we are oppressed. A journalist once exposed my identity as a person living with HIV without my permission, and I wanted to take legal action, but I couldn't. Even though I had legal grounds, I couldn't risk being exposed. If I were to go to court, as an HIV positive individual, would the judge even be on my side?”* **(P20, Male, 18-25 years old, Kuwait)**

Some participants noted that specific rules and regulations within GCC communities perpetuate stigma. For example, legal requirements that mandate confidentiality for PLHIV were perceived not as protective, but as implicitly reinforcing the idea that HIV is shameful. These policies, rather than promoting inclusion, were viewed by some as contributing to stigma by suggesting that a diagnosis should be hidden from others, including healthcare providers.

“*Unfortunately, there isn't enough awareness about HIV in our society. HIV has a very negative image in society. This is evidenced by the fact that the government requires PLHIV to keep their diagnosis confidential from doctors. If society had better awareness, PLHIV would not be afraid to disclose their diagnosis.”* **(P20, Male, 18-25 years old, Kuwait)**

The deportation of expatriates diagnosed with HIV/AIDS from GCC countries was viewed negatively by some GCC nationals living with HIV. Participants saw this practice as reinforcing stigma and contributing to negative perceptions about the condition. Deportation was interpreted as a symbol of the broader lack of acceptance of people living with HIV/AIDS in the region. It was seen as inconsistent with the realities of living a healthy life with HIV.

“*Why does the government deport any expat with HIV? If it's not a deadly disease, why are expats deported? … How are we expected to accept HIV if our government doesn't?”* **(P20, Male, 18-25 years old, Kuwait)**

### Future prospects of people living with HIV

Participants who are living with HIV/AIDS shared that they face significant challenges in securing employment. This has profoundly impacted their future aspirations, making it increasingly difficult for them to pursue their desired careers, resulting in the abandonment of lifelong dreams.

“*If I want to live normally, I must go live in the West. PLHIV can work any job there. They can work in restaurants, the military, and even as nurses. If the GCC governments want to convince us that they think we have rights, then why prevent us from working our dream jobs?*” **(P20, Male, 18-25 years old, Kuwait)**

In response to the detrimental impact of social stigma on PLHIV, they called for the implementation of legislative measures aimed at safeguarding their rights and facilitating the opportunity to lead a life that is meaningful and fulfilling. This includes ensuring equal access to healthcare services and employment opportunities.

“*The government must sue companies that refuse to employ PLHIV. They must be held accountable for their discrimination. They need to criminalise the discrimination.”* **(P20, Male, 18-25 years old, Kuwait)**

People living with HIV/AIDS expressed fear of being exposed and shamed when disclosing their diagnosis to a potential spouse. Marrying within the GCC community was seen as particularly challenging, as potential partners were perceived to hold the same stigmatizing views common in the region. Participants also raised concerns about the lack of confidentiality, noting that sharing a diagnosis with one person would likely lead to broader exposure due to the close-knit nature of GCC societies.

“*We are part of a Middle Eastern community where everyone knows everyone, so I'm terrified they'll expose me or gossip about me. Not a single person in my life knows [that I have HIV], none...”* **(P20, Male, 18-25 years old, Kuwait)**

## Discussion

This study revealed pervasive stigma surrounding HIV/AIDS across GCC societies, shaped by religious and moral judgments, misinformation, and negative portrayals in the media. PLHIV reported profound social exclusion, fear of disclosure, and discrimination within healthcare settings, which in some cases led to avoidance of care and deterioration of mental health. Participants reported facing challenges regarding marital prospects and employment. They highlighted the limited safe spaces for support, which are frequently limited to anonymous interactions via social media. While many advocated for raising awareness and involving PLHIV in stigma reduction efforts, others expressed doubt about the potential for societal change due to entrenched cultural attitudes.

As with many Muslim-majority communities, many participants denied the existence of HIV in their countries (23). The overwhelming response from Muslim communities has been to deny the existence of HIV, adopting a “not-in-this-region” and “it is still not our problem” approach (23–26). This denial not only heightens vulnerability to HIV transmission but also disregards the human rights of PLHIV, as the issue is not even recognized (23, 27).

There was a general sense of religious and moral immunity against HIV among participants. Such perceptions align with previous research showing that strong religious beliefs often result in a lower perceived risk of infection (12, 20, 21, 28). Many Muslims regard adherence to religious values as a primary safeguard against STIs (12, 20, 21, 28). However, this false sense of protection is concerning, as it may lead to under-detection of HIV, delayed testing, and missed opportunities for early treatment and prevention. HIV testing rates among Muslims remain consistently lower than those in other religious groups, a factor believed to contribute to the continued spread of STIs in Muslim communities (29, 30).

Many participants reflected on the presence of HIV/AIDS in the GCC and often attributed it, or the behaviors associated with its transmission, to Western influence. Prominent religious scholars have argued that HIV/AIDS is a consequence of the Western sexual revolution and the promiscuity it promoted (12, 23, 26). As HIV prevalence rises among Muslim populations, Western countries are frequently blamed for exporting liberal values, through tourism, television, and the promotion of sexual rights and for contributing to the spread of the virus within the region (23, 28, 31, 32). This framing enables communities to distance themselves from behaviors deemed morally unacceptable, reinforcing the perception that HIV/AIDS is a foreign issue rather than a result of local practices. By externalizing blame, stigma toward key populations may be intensified, while open dialogue about local transmission patterns and effective prevention strategies becomes more difficult.

Although stigma is often rooted in religious views that condemn behaviors associated with HIV risk, such as extramarital sex and drug use, the impact of misinformation and lack of knowledge about HIV cannot be overstated (20, 28, 33). For instance, physicians in Saudi Arabia with a limited understanding of HIV/AIDS demonstrated significantly higher levels of stigma compared to those with a greater understanding of the condition (34). One of the most pervasive misconceptions is that HIV is inevitably a deadly disease (4, 13, 28). When combined with the false belief that the virus can be transmitted through casual contact, such as air or handshakes, these misconceptions foster fear and reinforce stigma toward PLHIV (4, 13, 28). Lack of knowledge is prevalent in Muslim communities, where discussions about sexual health and access to comprehensive sex education remain limited (4, 13, 28).

Policies and regulations across the GCC appear to reinforce stigma toward people living with HIV/AIDS. One notable example is the deportation of expats diagnosed with HIV (35), which sends a message that those with the condition are not welcome in society. Some employment opportunities for GCC nationals living with HIV are limited, as many health and military-related roles require HIV testing during pre-employment screening, often resulting in exclusion from the workforce (36). Legal frameworks mandating confidentiality, while intended to protect individuals, were perceived by participants living with HIV as contributing to stigma by implying that HIV is a shameful condition that must be hidden. Rather than promoting inclusion, these policies often deepen social marginalization and hinder the full participation of people living with HIV/AIDS in public life.

Participants living with HIV described profound experiences of social exclusion, fear of disclosure, and internalized stigma. Previous literature has echoed these sentiments, with PLHIV describing stigma as worse than the disease itself (37). Many participants in this study viewed HIV as a form of divine punishment, reinforcing the widespread belief that individuals living with the virus are to blame for their condition. Stigma and entrenched taboos surrounding HIV/AIDS have been consistently reported across the MENA region (38, 39). Such negative perceptions are not limited to the general public but are also found among healthcare professionals in Muslim-majority contexts (39, 40). Prejudice, judgment, and mistreatment by healthcare providers have been identified as key barriers that discourage individuals at risk from seeking testing and treatment (12, 41, 42). Discriminatory attitudes among healthcare providers, often shaped by religious views on behaviors associated with risk, such as extramarital sex and drug use, can negatively affect the quality of care offered (12, 28). These findings are consistent with our study, in which participants living with HIV described significant challenges in accessing non-judgmental and comprehensive healthcare services due to persistent stigma and fear of exposure.

## Strengths and limitations

This study is among the first to qualitatively explore both public perceptions and the lived experiences of PLHIV in the GCC. The inclusion of participants from multiple GCC countries offered a broad understanding of HIV-related perceptions and attitudes. The use of qualitative interviews enabled the exploration of nuanced perspectives, particularly around stigma, gendered perceptions, and religious influences, which are underexamined in this context. Another notable strength is the inclusion of PLHIV, whose voices are rarely represented in GCC-based research. Their accounts and experiences added critical depth to the analysis and highlighted the real-life consequences of stigma, policy, and healthcare discrimination.

However, the sample of PLHIV was limited to two participants, which restricts the ability to generalize their experiences across all GCC countries. Social desirability bias may have influenced responses, particularly given the sensitive nature of the topic and prevailing cultural norms. Additionally, the recruitment methods may have led to a self-selection bias, attracting individuals who are more comfortable or open to discussing HIV-related topics. Despite these limitations, the findings provide valuable insights and lay the groundwork for future research and policy efforts aimed at improving awareness, inclusivity, and the quality of life for PLHIV in the GCC.

## Practice, research, and policy implications

This study is among the first in the GCC to qualitatively explore the lived experiences of people living with HIV, rather than focusing solely on public perceptions. The findings highlight the urgent need for more research that centers the voices of individuals living with HIV in Muslim communities, where stigma and social exclusion remain deeply rooted. Despite significant advancements in HIV treatment and management, which have enabled individuals to lead long and healthy lives, those living with HIV in the region continue to face widespread ostracization, discrimination, and fear of exposure (38, 39). Future research should build on these insights to develop strategies that improve the quality of life for PLHIV socially, emotionally, and structurally, and inform culturally appropriate interventions and policies that promote inclusion, dignity, and equity. Enhancing their experiences requires raising awareness and addressing structural and policy drivers of stigma and exclusion.

Participants emphasized the need for inclusive, ongoing awareness campaigns in schools, healthcare, and media to normalize HIV as manageable and uphold the rights of those affected. Involving PLHIV in these efforts can humanize the disease and challenge stereotypes. The need for inclusive, sustained awareness campaigns aligns with evidence from diverse global contexts showing that community-based, context-sensitive education efforts can improve social acceptance of PLHIV, correct misconceptions, and increase HIV testing (43–45). Involving PLHIV in these initiatives can further humanize the condition and counteract stereotypes that fuel stigma.

Addressing misinformation through targeted, culturally grounded education is essential to reducing HIV-related stigma, particularly in Muslim-majority societies where comprehensive sex education is limited (4, 13, 28). Participants frequently interpreted HIV risk through moral or religious frameworks, reinforcing perceptions of immunity and reducing motivation for testing. Incorporating targeted awareness efforts that challenge these assumptions and emphasize the importance of testing regardless of perceived moral standing is therefore critical.

While expanding awareness is essential, it cannot achieve lasting change without structural support. As such, policy reforms are needed to promote inclusivity and prevent discrimination. Healthcare policies should enforce accountability to prevent provider prejudice from harming patient care. Confidentiality policies must be framed as rights protections, not shame signals. Media should shift from fear-based HIV portrayals to accurate, humanizing narratives. Outreach should clarify transmission routes and promote prevention to combat misconceptions.

Integrating HIV services into primary healthcare has the potential to reduce stigma and improve access to care. By embedding HIV-related services within the broader health system, these interventions can help normalize HIV as part of routine healthcare rather than treating it as a separate, stigmatized issue. Evidence from other settings supports this approach (43, 46–49). In Thailand, incorporating HIV services into mainstream healthcare and embedding stigma-reduction strategies within the National AIDS Strategic Plan, such as anti-discrimination policies and routine stigma monitoring, significantly improved access and reduced stigma (46). Similarly, studies from sub-Saharan Africa found that integrating HIV services into primary healthcare settings led to increased service uptake and decreased stigma by positioning HIV as a routine health condition rather than an isolated disease (47–49). Such integrated models could be adapted for GCC countries to help tackle structural stigma, improve the healthcare experience for PLVIH, and promote their inclusion in society. These examples highlight the importance of multi-level approaches that combine education, healthcare reform, and inclusive policy to meaningfully reduce stigma and improve the quality of life for PLHIV.

## Conclusion

This study highlights the pervasive stigma surrounding HIV/AIDS in the GCC, shaped by religious and moral beliefs, misinformation, gender norms, and exclusionary policies. PLHIV face social isolation, fear of disclosure, and barriers to healthcare and employment. While participants called for greater awareness and involvement of PLHIV in advocacy, stigma remains deeply embedded in societal and institutional structures. As one of the first qualitative studies in the region to explore the lived experiences of people with HIV, these findings underscore the urgent need for inclusive policy reform, integrated stigma-reduction strategies, and further research centered on the voices of affected individuals.

## Data Availability

The datasets generated and analyzed during the current study are not publicly available due to the lack of consent to share raw material, but parts of the material can be available from the corresponding author upon reasonable request. Requests to access the datasets should be directed to samalageel@ksu.edu.sa.

## References

[1] WHO. HIV Statistics, Globally and by WHO Region, 2024. Geneva: WHO (2024).

[2] UNAIDS. 2024 Global AIDS Report—The Urgency of Now: AIDS at a Crossroads. Geneva: UNAIDS (2024).

[3] UNAIDS. Middle East and North Africa Regional Profile-−2024 Global AIDS Update The Urgency of Now: AIDS at a Crossroads. Geneva: UNAIDS (2024).

[4] Alomair N Alageel S Davies N Bailey JV. Sexually transmitted infection knowledge and attitudes among Muslim women worldwide: a systematic review. Sex Reprod Health Matters. (2020) 28:1731296. doi: 10.1080/26410397.2020.1731296 32202220 PMC7888124

[5] Arias-Colmenero T Pérez-Morente MÁ Ramos-Morcillo AJ Capilla-Díaz C Ruzafa-Martínez M Hueso-Montoro C . Experiences and attitudes of people with HIV/AIDS: A systematic review of qualitative studies. Int J Environ Res Public Health. (2020) 17:639. doi: 10.3390/ijerph17020639 31963822 PMC7014086

[6] Mumtaz GR Chemaitelly H AlMukdad S Osman A Fahme S Rizk NA . Status of the HIV epidemic in key populations in the Middle East and north Africa: knowns and unknowns. Lancet HIV. (2022) 9:e506–16. doi: 10.1016/S2352-3018(22)00093-5 35777412

[7] Rzeszutek M Gruszczyńska E Pieta M Malinowska P. HIV/AIDS stigma and psychological wellbeing after 40 years of HIV/AIDS: a systematic review and meta-analysis. Eur J Psychotraumatol. (2021) 12:1990527. doi: 10.1080/20008198.2021.1990527 34868481 PMC8635597

[8] Rueda S Mitra S Chen S Gogolishvili D Globerman J Chambers L . Examining the associations between HIV-related stigma and health outcomes in people living with HIV/AIDS: a series of meta-analyses. BMJ Open. (2016) 6:e011453. doi: 10.1136/bmjopen-2016-011453 27412106 PMC4947735

[9] Earnshaw VA Chaudoir SR. From conceptualizing to measuring HIV stigma: a review of HIV stigma mechanism measures. AIDS Behav. (2009) 13:1160–77. doi: 10.1007/s10461-009-9593-3 19636699 PMC4511707

[10] Turan B Hatcher AM Weiser SD Johnson MO Rice WS Turan JM . Framing mechanisms linking HIV-related stigma, adherence to treatment, and health outcomes. Am J Public Health. (2017) 107:863–9. doi: 10.2105/AJPH.2017.303744 28426316 PMC5425866

[11] Awaidy SA Ghazy RM Mahomed O. Progress of the gulf cooperation council (gcc) countries towards achieving the 95-95-95 UNAIDS targets: a review. J Epidemiol Glob Health. (2023) 13:397–406. doi: 10.1007/s44197-023-00097-1 37079171 PMC10116479

[12] Alomair N Alageel S Davies N Bailey JV. Muslim women's perspectives on the barriers to sexually transmitted infections testing and diagnosis in Saudi Arabia. Front Public Health. (2023) 11:1248695. doi: 10.3389/fpubh.2023.1248695 37881344 PMC10595004

[13] Aldhaleei WA Bhagavathula AS. HIV/AIDS-knowledge and attitudes in the Arabian Peninsula: a systematic review and meta-analysis. J Infect Public Health. (2020) 13:939–48. doi: 10.1016/j.jiph.2020.04.002 32359925

[14] UNAIDS. The Path that Ends AIDS: UNAIDS Global AIDS Update Univ. S C. Dep. Music 2023 (2023).

[15] Musheke M Ntalasha H Gari S Mckenzie O Bond V Martin-Hilber A . A systematic review of qualitative findings on factors enabling and deterring uptake of HIV testing in Sub-Saharan Africa. BMC Public Health. (2013) 13:220. doi: 10.1186/1471-2458-13-220 23497196 PMC3610106

[16] Apinundecha C Laohasiriwong W Cameron MP Lim S. A community participation intervention to reduce HIV/AIDS stigma, Nakhon Ratchasima province, northeast Thailand. AIDS Care. (2007) 19:1157–65. doi: 10.1080/09540120701335204 18058400

[17] Willis JW Jost M Nilakanta R. Foundations of Qualitative Research: Interpretive and Critical Approaches. Thousand Oaks, CA: Sage Publications (2007).

[18] Ritchie J Lewis J McNaughton Nicholls C Ormston R. Qualitative Research Practice: A Guide for Social Science Students and Researchers. Thousand Oaks, CA: Sage Publications (2013).

[19] Green J Thorogood N. Qualitative Methods for Health Research. Thousand Oaks, CA: Sage Publications (2018).

[20] Alageel S Alomair N. Are the Arab Gulf States ready for HIV/AIDS discussions? A qualitative thematic analysis. Sex Res Soc Policy. (2023) 1–10. doi: 10.1007/s13178-023-00840-0

[21] Alageel S Alsadhan NM Alkhaldi G Alkasabi R Alomair N. Public perceptions of HIV/AIDS awareness in the Gulf Council Cooperation countries: a qualitative study. Int J Equity Health. (2024) 23:269. doi: 10.1186/s12939-024-02346-6 39695658 PMC11656960

[22] Braun V Clarke V. Reflecting on reflexive thematic analysis. Qual Res Sport Exerc Health. (2019) 11:589–97. doi: 10.1080/2159676X.2019.1628806

[23] Hamidi A Regmi P van Teijlingen E. Islamic perspectives on HIV: a scoping review. Discov Soc Sci Health. (2024) 4:6. doi: 10.1007/s44155-024-00063-7

[24] Speakman S. Comparing the impact of religious discourse on HIV/AIDS in Islam and Christianity in Africa. Vanderb Undergrad Res J. (2012) 8:3490. doi: 10.15695/vurj.v8i0.3490

[25] Abu-Moghli F Nabolsi M Khalaf I Suliman W. Islamic religious leaders' knowledge and attitudes towards AIDS and their perception of people living with HIV/AIDS: a qualitative study. Scand J Caring Sci. (2010) 24:655–62. doi: 10.1111/j.1471-6712.2009.00757.x 20409060

[26] Maoulidi S. Muslim women responding to HIV/AIDS in Tanzania. J Muslim Minor Aff. (2003) 23:375–9. doi: 10.1080/1360200032000139992

[27] Monshipouri M Trapp T. HIV/AIDS, religion, and human rights: a comparative analysis of Bangladesh, Indonesia, and Iran. Hum Rights Rev. (2012) 13:187–204. doi: 10.1007/s12142-011-0213-z

[28] Alomair N Alageel S Davies N Bailey J. Muslim women's knowledge, views, and attitudes towards sexually transmitted infections in Saudi Arabia: a qualitative study. PLoS ONE. (2023). doi: 10.1371/journal.pone.0286822 37352200 PMC10289450

[29] Abu-Raddad L Akala FA Semini I Riedner G Wilson D Tawil O . Characterizing the HIV/AIDS epidemic in the Middle East and North Africa: Time for Strategic Action. Washington, DC: The World Bank (2010).

[30] Hearld KR Wu D Budhwani H. HIV testing among Muslim women in the United States: results of a National Sample Study. Health Equity. (2021) 5:17–22. doi: 10.1089/heq.2020.0041 33564736 PMC7868576

[31] Maulana AO Krumeich A Van Den Borne B. Emerging discourse: Islamic teaching in HIV prevention in Kenya. Cult Health Sex. (2009) 11:559–69. doi: 10.1080/13691050902792771 19437176

[32] Becker F. The virus and the scriptures: Muslims and AIDS in Tanzania. J Relig Afr. (2007) 37:16–40. doi: 10.1163/157006607X166573

[33] Fatoki B. Understanding the Causes and Effects of Stigma and Discrimination in the Lives of HIV People Living with HIV/AIDS: qualitative study. J AIDS Clin Res. (2016) 7:635. doi: 10.4172/2155-6113.1000635

[34] Memish ZA Filemban SM Bamgboyel A Al Hakeem RF Elrashied SM Al-Tawfiq JA . Knowledge and attitudes of doctors toward people living with HIV/AIDS in Saudi Arabia. J. Acquir Immune Defic Syndr. (2015) 69:61–7. doi: 10.1097/QAI.0000000000000550 25642972

[35] UNAIDS. Still not Welcome—HIV-Related Travel Restrictions. Geneva: UNAIDS (2019).

[36] The Bureau of Experts at the Council of Ministers. المكتسب المناعي العوز متلازمة من: الوقاية ظام اجباتهم وو المصابين وحقوق)الإيدز( [The System for the prevention of acquired immune deficiency syndrome (AIDS) and the rights and duties of the infected]. Riyadh: Bureau of Experts at the Council of Ministers (2018). Arabic.

[37] Gruszczyńska E Rzeszutek M. HIV/AIDS stigma accumulation among people living with HIV: a role of general and relative minority status. Sci Rep. (2023) 13:10709. doi: 10.1038/s41598-023-37948-7 37400505 PMC10318048

[38] Roudi-Fahimi F. Time to intervene: preventing the spread of HIV/AIDS in the Middle East and North Africa. Population Reference Bureau. Washington, DC: Population Reference Bureau (PRB) (2007), 1–8.

[39] Gökengin D Doroudi F Tohme J Collins B Madani N. HIV/AIDS: trends in the Middle East and North Africa region. Int J Infect Dis. (2016) 44:66–73. doi: 10.1016/j.ijid.2015.11.008 26948920

[40] Alawad M Alturki A Aldoghayyim A Alrobaee A Alsoghair M. Knowledge, attitudes, and beliefs about HIV/AIDS and people living with HIV among medical students at Qassim University in Saudi Arabia. Int J Health Sci. (2019) 13:22. doi: 10.12816/0055171 31501649 PMC6728128

[41] Schwarcz S Richards TA Frank H Wenzel C Hsu LC Chin CJ . Identifying barriers to HIV testing: personal and contextual factors associated with late HIV testing. AIDS Care. (2011) 23:892–900. doi: 10.1080/09540121.2010.534436 21424942

[42] Deblonde J De Koker P Hamers FF Fontaine J Luchters S Temmerman M. Barriers to HIV testing in Europe: a systematic review. Eur J Public Health. (2010) 20:422–32. doi: 10.1093/eurpub/ckp231 20123683

[43] Stangl AL Lloyd JK Brady LM Holland CE Baral S. A systematic review of interventions to reduce HIV-related stigma and discrimination from 2002 to 2013: how far have we come? J. Int AIDS Soc. (2013) 16:18734. doi: 10.7448/IAS.16.3.18734 24242268 PMC3833106

[44] Dessie ZG Zewotir T. HIV-related stigma and associated factors: a systematic review and meta-analysis. Front Public Health. (2024) 12:1356430. doi: 10.3389/fpubh.2024.1356430 39109161 PMC11300231

[45] Kimera E Alanyo LG Pauline I Andinda M Mirembe EM. Community-based interventions against HIV-related stigma: a systematic review of evidence in Sub-Saharan Africa. Syst Rev. (2025) 14:8. doi: 10.1186/s13643-024-02751-6 39794796 PMC11720753

[46] Siraprapasiri T Srithanaviboonchai K Chantcharas P Suwanphatthana N Ongwandee S Khemngern P . Integration and scale-up of efforts to measure and reduce HIV-related stigma: the experience of Thailand. AIDS. (2020) 34:1279–87. doi: 10.1097/QAD.0000000000002586 32881799

[47] Goldstein D Salvatore M Ferris R Phelps BR Minior T. Integrating global HIV services with primary health care: a key step in sustainable HIV epidemic control. Lancet Global Health. (2023) 11:e1120–4. doi: 10.1016/S2214-109X(23)00156-0 37349037

[48] Odeny TA Penner J Lewis-Kulzer J Leslie HH Shade SB Adero W . Integration of HIV care with primary health care services: effect on patient satisfaction and stigma in rural Kenya. AIDS Res Treat. (2013) 2013:485715. doi: 10.1155/2013/485715 23738055 PMC3664481

[49] Dzinamarira T Rwibasira G Mwila L Moyo E Mangoya D Moyo P . Advancing sustainable HIV services through integration in primary healthcare in Sub-Saharan Africa: a perspective on practical recommendations. Healthcare. (2025) 13:192. doi: 10.3390/healthcare13020192 39857218 PMC11764692

